# Supplementary figures and images for: ER-Ca2+ sensor STIM regulates neuropeptides required for development under nutrient restriction in Drosophila
Source: PLoS One. 2019 Jul 11;14(7):e0219719. doi: 10.1371/journal.pone.0219719 (PMC6622525; doi:10.1371/journal.pone.0219719)

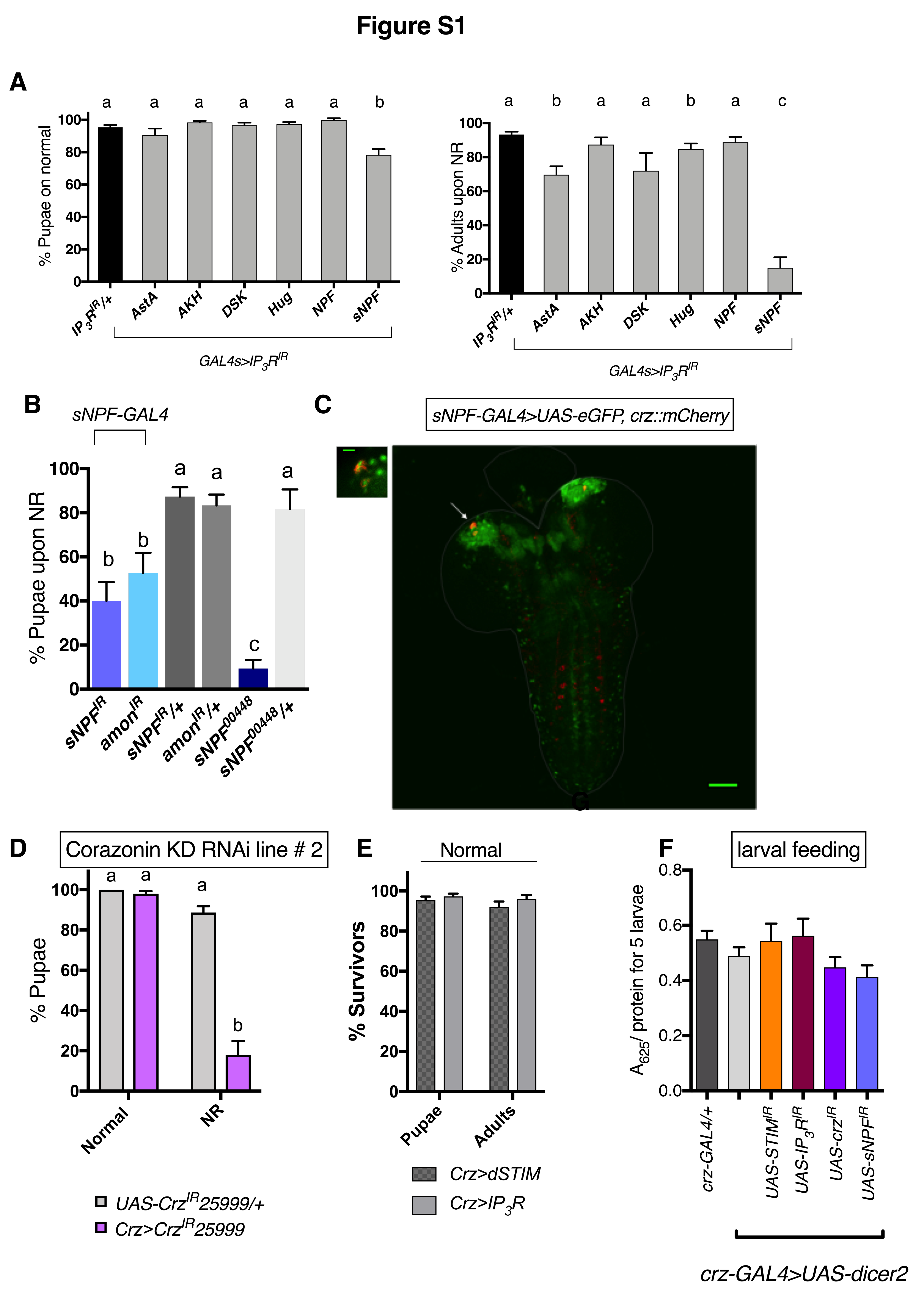

Supplement: S1 Fig — A. Genetic Screen. GAL4s for various peptides were used to drive IP3RIR and the corresponding larvae were tested for their ability to pupariate on normal or NR media. AstA: Allatostatin A; AKH: Adipokinetic Hormone; DSK: Drosulphakinin; Hug: Hugin; NPF: Neuropeptide F; sNPF: short Neuropeptide F. B. % Pupae in NR media when sNPF is reduced by RNAi (sNPRIR) or by reducing an enzyme required for neuropeptide processing (amonIR) in sNPF-GAL4 expressing cells, or in a hypomorphic sNPF mutant (sNPF00448). C. 3rd instar larval brain expressing GFP in sNPF-GAL4 producing neurons and a mcherry-tagged corazonin (Crz::mcherry). Note the co-localisation of sNPF-GAL4 with Crz::mcherry, only in the brain lobes (arrow). D. % Pupae on normal and NR media when Crz is reduced in Crz+ neurons, using a second RNAi line (crzIR25999) E. % Pupae and adults that developed on normal food from larvae where Crz+ neurons over-expressed either dSTIM or IP3R. Differences are not statistically significant. F. Food intake as measured by absorbance of coloured food (A625) fed to larvae for the indicated genotypes. N = 8 sets of 5 larvae each. Differences are not statistically significant. one-way ANOVA with a post hoc Tukey’s test p<0.05 for (A), (B), (F). Ordinary two-way ANOVA with Sidak’s multi-comparison test p<0.05 for (D). Bars with the same alphabet represent statistically indistinguishable groups. Data represents mean ± SEM. (TIFF) [file pone.0219719.s001.tiff]

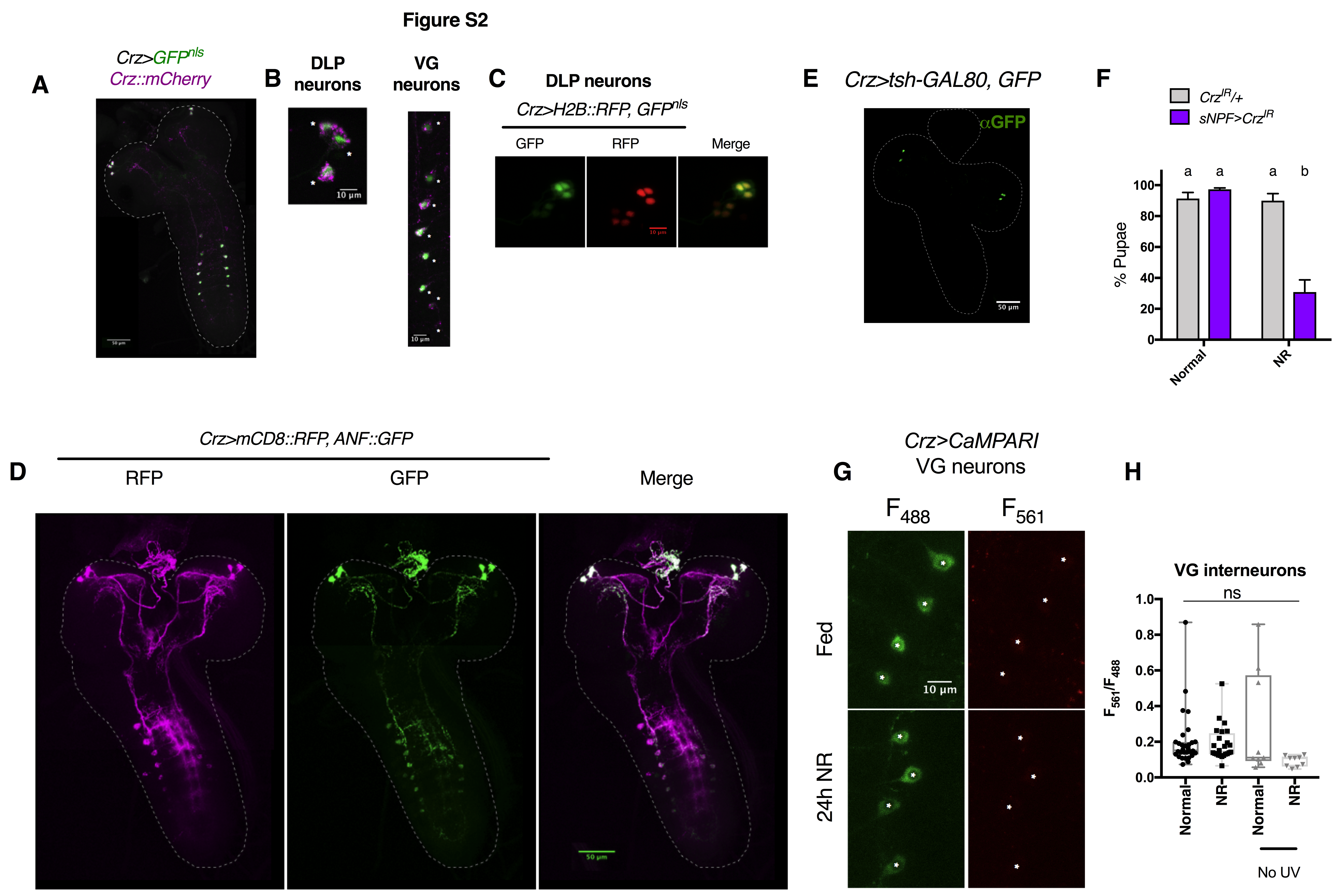

Supplement: S2 Fig — A. and B. Expression pattern of Crz-GAL4 (observed via GFPnls) closely matched the expression of Crz (observed by a genomically integrated, mCherry-tagged corazonin: Crz::mCherry). C. Crz-GAL4 also expresses in ~4–5 additional neurons in the DLP region, that are not marked by Crz::mCherry (See A). The level of expression also varies with the marker. GFPnls vs histone-tagged to RFP (H2B::RFP).D. Overall distribution of NPs in Crz+ neurons followed by the expression of GFP-tagged rat ANF (ANF::GFP) and mCD8-tagged membrane bound RFP (UAS-mRFP). Note the exclusion of ANF from projections that end in the SEZ. E. Representative image. Expression of tsh-GAL80 in crz>GFP expressing brains causes the loss of GFP expression in the VG. F. % Pupae when Corazonin is reduced (CrzIR) in sNPF-GAL4 expressing neurons. This allows restricted expression of CrzIR only in DLPs (S1C Fig). Ordinary two-way ANOVA with a post hoc Sidak’s multi-comparison test p<0.05. Data represents mean ± SEM.G. Representative image. Expression of the UV-activated Ca2+ indicator, CaMPARI in Crz+ VG neurons. F561 reflects Ca2+ levels, while F488 reflects levels of the indicator. H. Quantification of F561/488 ratio in the presence and absence of UV-stimulation, after 24hrs in either normal or NR food, in Crz+ VG neurons. N>7 larvae for UV-stimulated; N = 3 for No UV stimulation. Kruskal-Wallis Test with Dunn’s multi-comparison correction p<0.05. Bars with the same alphabet represent statistically indistinguishable groups. (TIFF) [file pone.0219719.s002.tiff]

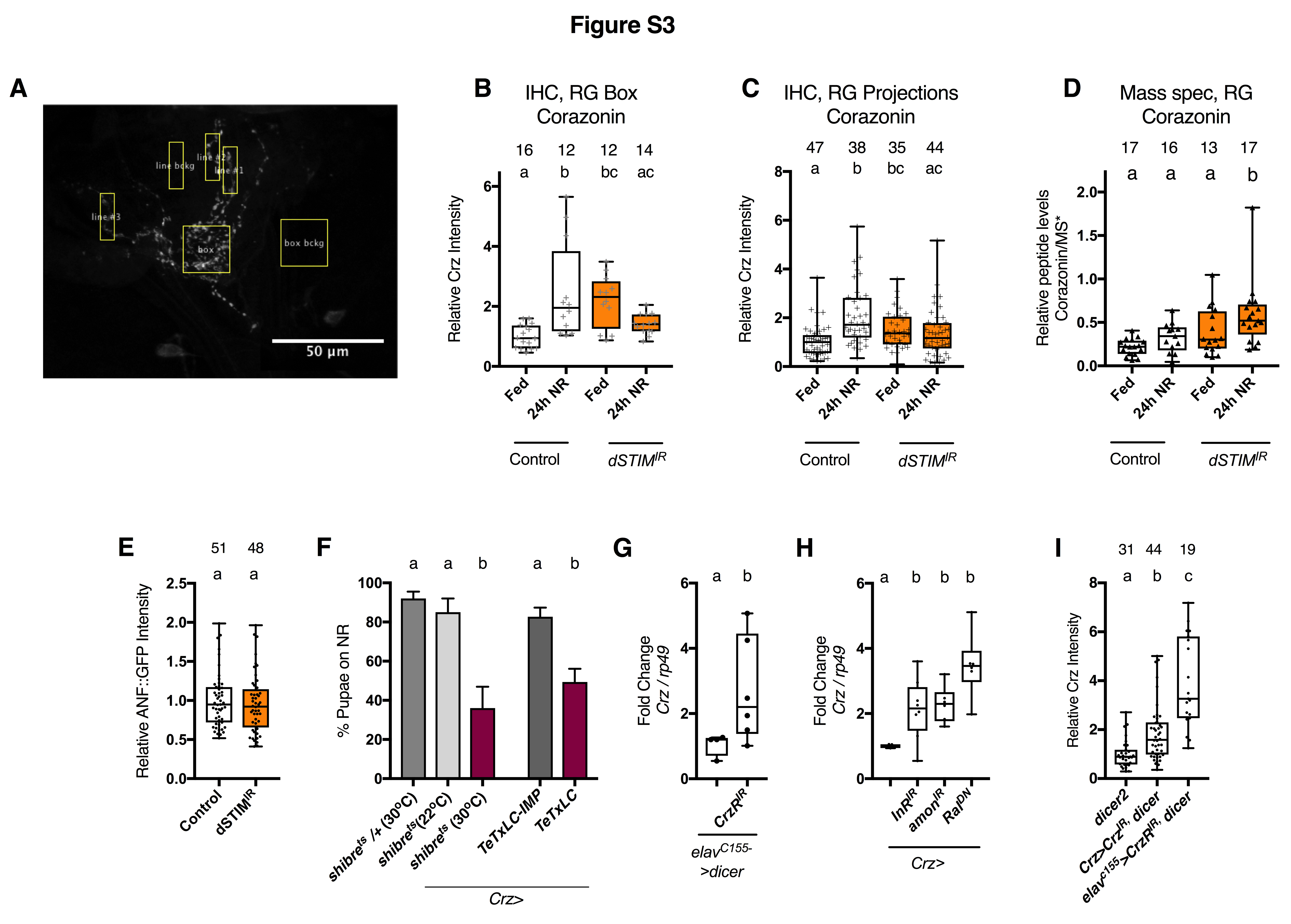

Supplement: S3 Fig — A. Representative image. How “Box” and “projection” areas were delineated for measuring levels of Crz by immunofluorescence, at the ring gland (RG). Box is a 50X50 px square. Lines are a 50X15px rectangle. These measurements were made in a subset of DLPs for which Crz intensity were measured in cell bodies, plotted in Fig 3A. B. and C. Quantification of Crz levels at the RG. D. Relative Crz peptide levels measured on dissected RGs (N atop bars) and quantified using MALDI-MS. Externally added heavy standard (MS*) was used to normalise peptide levels between samples. Kruskal-Wallis Test with Dunn’s multi-comparison correction p<0.05 E. Relative levels of the Rat Neuropeptide ANF::GFP measured by GFP fluorescence, in Crz+ DLPs in either control (crz>dicer2) or dSTIMIR condition (crz>dstimIR,dicer2). Two-tailed t-test. Total number of cell bodies counted mentioned atop bars. N>15 brains. F. % Pupae when vesicle release is perturbed either by expressing a dynamin mutant activated at 30°C (Shibrets), or tetanus toxin light chain (TeTxLc). TeTxLC-IMP: inactivated TeTxLc. Ordinary one-way ANOVA with a post hoc Tukey’s test p<0.05. Data represents mean ± SEM. G. Crz mRNA levels in larval brain with pan-neuronal reduction in CrzR (crzRIR). Two-tailed t-test. N = 4. H. Crz mRNA levels in larval brains where protein synthesis (InRIR) or peptide processing (amonIR) or vesicle exocytosis (RalDN) are perturbed in Crz+ neurons. Ordinary one-way ANOVA with a post hoc Tukey’s test p<0.05. N>4. I. Relative levels of Crz measured on cell bodies of Crz+ DLP neurons when either Crz (CrzIR) is reduced in Crz+ neurons or its receptor CrzR (crzRIR) is reduced pan-neuronally. Kruskal-Wallis Test with Dunn’s multicomparison correction p<0.05. N>10 brains. Bars with the same alphabet represent statistically indistinguishable groups. (TIFF) [file pone.0219719.s003.tiff]

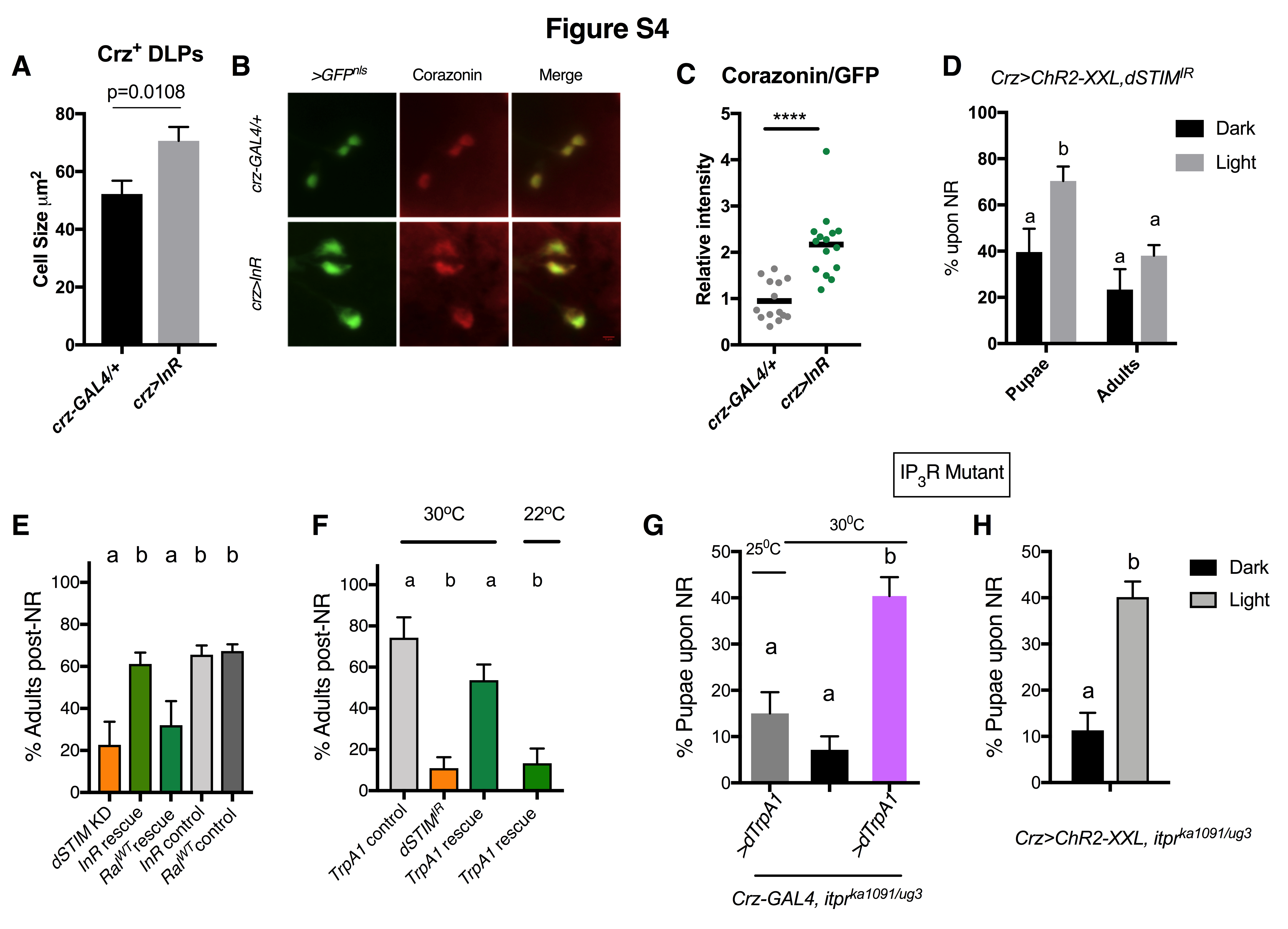

Supplement: S4 Fig — A. Cell size of DLP neurons, upon over-expression of Insulin receptor (InR) in Crz+ neurons. N = 5 brains. Student’s t-test. Representative images in B. C. Relative Crz peptide levels in cell bodies of Crz+ DLP neurons expressing InR. N = 5. Student’s t-test. p<0.001 D. % Pupae upon NR, when in Crz+ neurons, dSTIM was reduced, and ChR2-XXL, the light activated channel was ectopically expressed. Larvae were reared in the dark and post-transfer to NR, either continued to be kept in the “Dark” or moved to an incubator with 24 lights (white light) ON (Light) till the end of the pupariation assay (~ 10days). two-way ANOVA with a post hoc Sidak’s multi-comparison test p<0.05. E. % Adults recovered upon over-expression of Insulin receptor (InR) or Ral (RalWT) in Crz+ neurons expressing dSTIMIR. InR rescue: crz>InR,dSTIMIR,dicer2. RalWT rescue: crz> RalWT, dSTIMIR,dicer2. InR control: dicer2;InR/+; dSTIMIR /+;. RalWT control: RalWT /+; dSTIMIR /+. Ordinary one-way ANOVA with a post hoc Tukey’s test p<0.05. F. % Adults recovered upon over-expression of TrpA1 in Crz+ neurons expressing dSTIMIR. TrpA1 control: dicer2;TrpA1/+; dSTIMIR /+; TrpA1 rescue: crz>TrpA1, dSTIMIR,dicer2 Ordinary one-way ANOVA with a post hoc Tukey’s test p<0.05. N = 6 Ordinary one-way ANOVA with a post hoc Tukey’s test p<0.05. N = 6. G and H. % Pupae upon NR, in an hypomorphic IP3R mutant (itprka1091/ug3) with Crz+ neurons over-expressing either dTrpA1, and post-transfer incubating at 30°C for 24hours, or ChR2-XXL and reared in light till the end of the assay. Ordinary one-way ANOVA with a post hoc Tukey’s test p<0.05. for (G). Student’s t-test for (H). Bars with the same alphabet represent statistically indistinguishable groups. Data represents mean ± SEM. (TIFF) [file pone.0219719.s004.tiff]
